# Supplementary material for: The interactome of intact mitochondria by cross-linking mass spectrometry provides evidence for coexisting respiratory supercomplexes
Source: Mol Cell Proteomics. 2017 Dec 8;17(2):216–32. doi: 10.1074/mcp.RA117.000470 (PMC5795388; doi:10.1074/mcp.RA117.000470)
Supplement: Supplemental Data [file supp_RA117.000470_133922_0_supp_23978_fzffwf.zip › spectra_annotation/mito_DR_spectra_annotation/389-1-3-1-4-1.pdf]

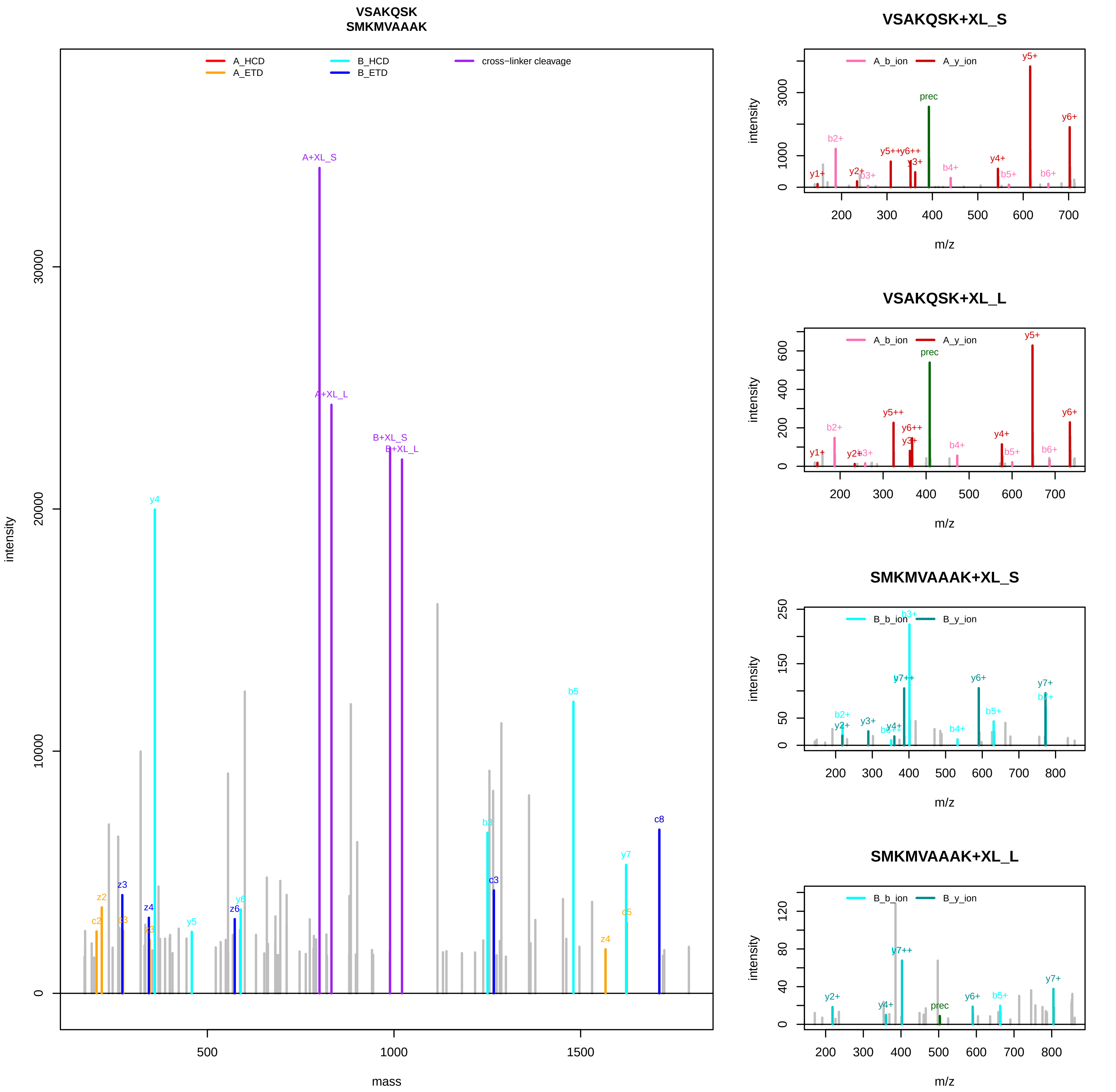
VSAKQSK
SMKMVAAAK
A_HCD
A_ETD
B_HCD
B_ETD
cross−linker cleavage
A+XL_S
A+XL_L
B+XL_S
B+XL_L
y4
b5
b3
c8
y7
c5
z4
c3
z6
y6
y5
z4
z3
z2
c2
c3
intensity
mass
VSAKQSK+XL_S
A_b_ion
A_y_ion
prec
y5+
y6+
b2+
y5++
y6++
y3+
y2+
b3+
y1+
b4+
y4+
b5+
b6+
intensity
m/z
VSAKQSK+XL_L
A_b_ion
A_y_ion
prec
y5+
y6+
y5++
y6++
y3+
b2+
y2+
b3+
y1+
b4+
y4+
b5+
b6+
intensity
m/z
SMKMVAAAK+XL_S
B_b_ion
B_y_ion
b3+
y7++
y6+
y7+
b7+
b2+
y2+
y3+
y4+
b6++
b4+
b5+
intensity
m/z
SMKMVAAAK+XL_L
B_b_ion
B_y_ion
y7++
y7+
y2+
y4+
prec
y6+
b5+
intensity
m/z
